# Increased H3K27ac level of ACE mediates the intergenerational effect of low peak bone mass induced by prenatal dexamethasone exposure in male offspring rats

**DOI:** 10.1038/s41419-018-0701-z

**Published:** 2018-05-29

**Authors:** Hao Xiao, Yinxian Wen, Zhengqi Pan, Yangfan Shangguan, Jun Qin, Yang Tan, Hongqiang Jiang, Bin Li, Qi Zhang, Liaobin Chen, Hui Wang

**Affiliations:** 1 Department of Orthopedic Surgery, Zhongnan Hospital of Wuhan University, Wuhan, 430071 China; 2 Hubei Provincial Key Laboratory of Developmentally Originated Disease, Wuhan, 430071 China; 3 Department of Pharmacology, Basic Medical School of Wuhan University, Wuhan, 430071 China

## Abstract

Prenatal dexamethasone exposure (PDE) induces developmental toxicities of multiple organs in offspring. Here, we verified the intergenerational effect of low peak bone mass induced by PDE and investigated its intrauterine programming mechanism. Pregnant rats were injected subcutaneously with 0.2 mg/kg/d dexamethasone from gestation day (GD) 9 to 20. Some pregnant rats were killed for the fetuses on GD20, and the rest went on to spontaneous labor to produce the first-generation (F1) offspring. The adult F1 male offspring were mated with normal females to produce the F2 offspring. In vivo, PDE leads to low peak bone mass in F1 male offspring rats at postnatal week (PW) 28. Furthermore, PDE reduced the bone mass in F1 male offspring from GD20 to PW12. Meanwhile, the osteogenic differentiation was suppressed and the local renin–angiotensin system (RAS) was activated continuously by PDE. Moreover, the histone 3 lysine 27 acetylation (H3K27ac) level in angiotensin-converting enzyme (ACE) promoter region was increased by PDE from GD20 to PW12. Likewise, PDE induced the low peak bone mass and the activated local RAS in F2 male offspring. Meaningfully, the H3K27ac level of ACE was increased by PDE in the F2 offspring. In vitro, dexamethasone inhibited bone marrow mesenchymal stem cells (BMSCs) osteogenic differentiation and promoted RAS activation. Furthermore, dexamethasone recruited CCAAT/enhancer-binding protein α and p300 into the BMSCs nucleus by activating glucocorticoid receptor, which cooperatively increased the H3K27ac level in the ACE promoter region. In conclusion, PDE induced the low peak bone mass and its intergenerational effect, which was mediated by sustained activation of RAS via increasing H3K27ac level of ACE.

## Introduction

Dexamethasone, as a kind of synthetic glucocorticoids, can promote fetal lung maturity, reduce neonatal respiratory distress syndrome, and decrease perinatal mortality^1,2^. Thus, it has been widely used as a medication for several types of severe disorders of pregnancy, including premature delivery^3,4^. However, prenatal dexamethasone treatment is associated with low birth weight, developmental toxicities of multiple organs and susceptibility to several kinds of diseases in adulthood^5,6^. Previous studies indicated that the application of dexamethasone during pregnancy or childhood could lead to bone dysplasia, which may diminish bone quality and mechanical properties^7–9^. Despite the known negative effects of prenatal dexamethasone exposure (PDE) on bone development, there appears to be a significant knowledge gap about the intergenerational effect of PDE on peak bone mass and its intrauterine programming mechanism.

Peak bone mass is the maximum bone mass in humans or animals under physiological conditions. Thus, it is a key determinant of skeletal health throughout life. In humans, the rate of bone mass accrual peaks during puberty between 11 and 14 years old (the span is equivalent to 6–12 weeks old of young rats), and they gain most of their peak bone mass at this period^10^. After adolescence, the bone density continues to increase slowly and peaks around the age of 20–35 years (the span is equivalent to 7–9 months of age in rats)^11^. The importance of maximizing bone mineral accrual during the period of bone development is clearly recognized^12^. A low peak bone mass will lead to a higher risk of osteoporosis, so the acquisition of peak bone mass is an essential factor in determining the future risk of osteoporosis and fractures^13,14^. Osteoblasts that differentiate from bone marrow mesenchymal stem cells (BMSCs) are the fundamental factor for the formation of bone mass through synthesis and secretion bone matrix^15–17^. Therefore, any factors that inhibit BMSCs differentiating osteoblasts may lead to the low peak bone mass and high susceptibility to osteoporosis in adulthood. The classic signaling pathway of renin–angiotensin system (RAS) is that angiotensin-converting enzyme (ACE) activates angiotensin I (Ang I) into Ang II; and the latter considered as the main effector peptide of the RAS plays specifically biological roles via specific receptors. In addition, the local RAS in different organs or tissues also plays an important role in diverse physiological functions including cell proliferation, differentiation, and apoptosis ^18–20^.

It has been demonstrated that a stimulus or insult occurring during critical periods of fetal growth and development can permanently alter tissue structure and function, which is termed “intrauterine programming”^21^. Indeed, evidence from both human and animal studies suggest that adult pathophysiology could be induced by prenatal adverse environment^22,23^. Epidemiological studies also show that this phenomenon was not limited to the first-generation (F1) offspring and that programming effects may persist in subsequent generations^24,25^. Furthermore, increasing studies indicated that the potential importance of epigenetic modifications in the intergenerational inheritance of the programming phenotype^26–28^. In this study, pregnant rats were treated with dexamethasone during middle and late pregnancy. By detecting the morphological index and functional gene expression in F1 and F2 male offspring, we aimed to confirm the low peak bone mass and its intergenerational effect induced by PDE. Moreover, we explored whether the intrauterine programming alteration of RAS mediated the low peak bone mass. This study would be helpful to uncover the long-term harmful effect on bone development in response to PDE and to find the early therapeutic target. Meanwhile, this study will provide experimental evidence and new academic perspectives to illuminate the theory of Developmental Origins of Health and Disease.

## Materials and methods

### Chemicals and reagents

Dexamethasone was obtained from the Shuanghe Pharmaceutical Company (Wuhan, China). The Captopril for ACE inhibitor, RU486 for glucocorticoid receptor (GR) inhibitor, C646 for p300 inhibitor, and Alizarin Red S were purchased from Sigma-Aldrich (St. Louis, MO, USA). The Ang II enzyme-linked immunosorbent assay (ELISA) kit, osteocalcin (OCN) assay kit and tartrate-resistant acid phosphatase (TRAP) assay kit were obtained from Jiancheng Bioengineering Institute (Nanjing, China). The nucleus and cytoplasm protein extraction kit was purchased from Beyotime Biotech Co., Ltd. (Shanghai, China) and the DNA purification kit was purchased from Tiangen Biotech Co., Ltd. (Beijing, China). The α-Minimum Essential Medium (MEM) was purchased from HyClone Co. (Logan, UT, United States) and fetal bovine serum was purchased from Gibco Co. (Detroit, MI, United States). TRIZOL was purchased from Invitrogen Co. (Carlsbad, CA, USA). Reverse transcription and real-time quantitative polymerase chain reaction (RT-qPCR) kits were purchased from Takara Biotech Co., Ltd. (Dalian, China). The SYBR Green dye was purchased from Applied Biosystems through Thermo Fisher scientific (ABI) (Foster City, CA, USA). All primers were synthesized by Sangon Biotech Co., Ltd. (Shanghai, China). The antibodies for ACE (ab11734), angiotensin receptor 1 (AT1R) (ab9391), AT2R (ab92445), CCAAT/enhancer-binding protein α (C/EBPα) (ab40764), and p300 (ab14984) were purchased from Abcam plc. (Cambridge, Cambridge shire, UK). The antibody for GR (sc-376426) was purchased from Santa Cruz Biotech Co. (Santa Cruz, CA, USA). The antibodies for histone 3 lysine 9 acetylation (H3K9ac) (A7255) and H3K27ac (A7253) were purchased from ABclonal Biotech Co., Ltd (Wuhan, China).

### Animals and treatment

**Fig. 1:**
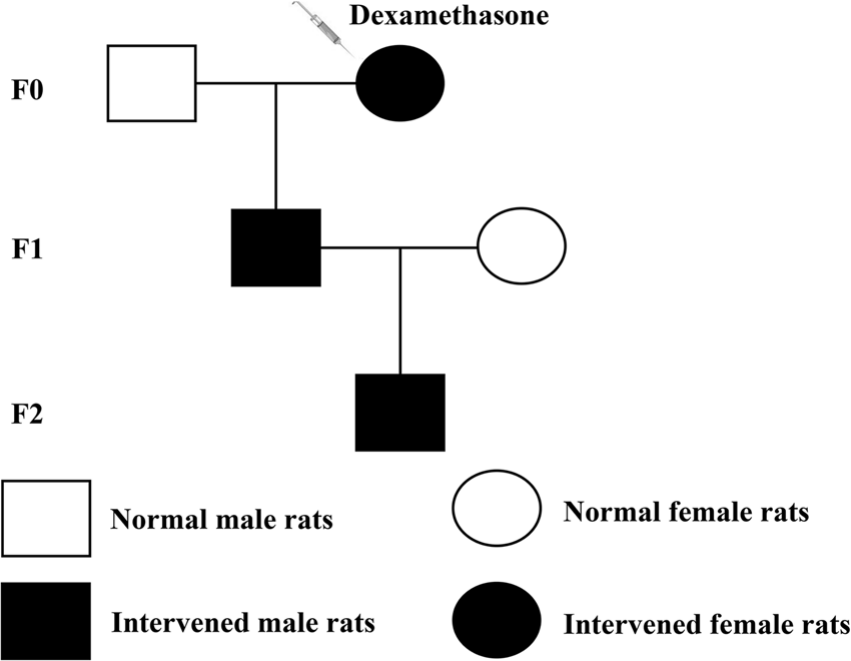
The animal experimental procedures

Animal experiments were complied with the Committee on the Ethics of Animal Experiments of the Wuhan University School of Medicine approved the protocol (Permit No. 201709) and performed in the Center for Animal Experiment of Wuhan University (Wuhan, China) according to the Guidelines for the Care and Use of Laboratory Animals of the Chinese Animal Welfare Committee. All animals were housed in temperature-controlled room (temperature: 18–22 °C; humidity: 40–60%; light cycle: 12 h light–dark cycle) and allowed free access to food and water. In order to reduce bias in animal experiments, rats were housed and treated by a technician, whereas different co-authors were in charge of bone sample harvesting and data analysis, respectively. The experimental procedures and treatment methods in this study were described as follows (Fig. 1).

F0: Healthy and specific pathogen-free Wistar rats weighing 209 ± 12 g (females) and 258 ± 17 g (males) were obtained from the Experimental Center of the Hubei Medical Scientific Academy (No. 2012–2014, certification number: 42000600002258, Hubei, China). All rats mentioned above were acclimated 1 week before experimentation, and two female rats were placed together with one male rat overnight in a cage for mating. On the next day, the appearance of sperm in vaginal smears confirmed mating, and the mating day was considered as gestation day (GD) 0. Then, pregnant rats were transferred to individual cages and randomly divided into the control and PDE groups. As the distinct condensation of the hindlimb bud in rodents appears around GD9^29^, so pregnant rats were injected subcutaneously with dexamethasone (0.2 mg/kg) or equal volume of saline every morning (between 08:00 and 09:00 a.m.) from GD9 to GD20. In order to obtain fetal bone samples in F1 offspring, some of the pregnant rats were killed after anesthesia with isoflurane and one male fetus was randomly selected from each litter on GD20 (*n* = 8 per group). The left femurs and tibias were collected and fixed with 4% paraformaldehyde overnight and then embedded in paraffin for histological and immunohistochemistry analysis, while the right femurs and tibias were frozen immediately in liquid nitrogen and then conserved at −80 °C refrigerator for further analysis.

F1: The rest pregnant rats including the control and PDE groups went on to spontaneous labor to produce the F1 offspring. The pregnant rats with litter size ≥ 10 were selected and the pups were normalized to 10; the male/female ratio was ~ 1:1. And the number of pregnant rats assigned to each group was eight. Three subgroups of the F1 male offspring were randomly selected and killed with isoflurane at postnatal week (PW) 2, PW12, and PW28 (*n* was set eight from eight different litters at each time point). The left femurs were dissected and fixed in 70% ethanol for micro-computed tomography (micro-CT) analysis. The left tibias were fixed with 4% paraformaldehyde and then embedded in paraffin for histological or immunohistochemistry analysis. The right femurs and tibias were frozen immediately in liquid nitrogen and then conserved at −80 °C refrigerator for further analysis.

F2: The F1 male offspring from the control and PDE groups in adulthood mated with normal female rats and avoid inbreeding to generate F2 offspring. At PW12, one male rat per litter in F2 offspring was randomly selected and killed as mentioned above (*n* = 8 per group). Then, their bone tissue was collected and underwent studies similar to those performed on F1 offspring.

### Histological and immunohistochemistry analysis

To explore morphology and calcification of hindlimb long bones in the fetal rats, serial longitudinal sections (5 μm thick) were cut and one out of six sections were stained with 5% AgNO_3_ until they become dark brown for von Kossa staining to quantify the bone mass in the primary ossification center (*n* = 8 per group). The primary indices include the mineralized area (Md.Ar) and bone perimeter (B.Pm) were measured on von Kossa-stained sections at × 100 magnification using Image-Pro Plus 6.0 as described previously^30^. For immunohistochemical analysis, the bone tissue slices were cut mentioned above at each time point (*n* = 8 per group). Then, these slices were dewaxed and washed in phosphate buffer saline (PBS). After antigen retrieval, bone tissue slices were blocked in 5% blocking serum at room temperature for 1 h, and incubated with a primary antibody for ACE (1:20 dilution) overnight at 4 °C. After washing with PBS, slices were incubated with a biotinylated secondary antibody and then with an avidin-biotinylated horseradish peroxidase complex solution according to the manufacturer’s directions. Finally, peroxidase activity was determined with a Diaminobenzidine staining kit. For negative controls in immunohistochemistry, parallel slices were immunostained with non-immune rabbit IgG as a primary antibody. The intensity of immunostaining was determined by measuring the mean optical density in six sections from different samples and using the Photo Imaging System (Nikon H550S, Japan).

### Micro-CT measurement

The left femurs dissected from F1 male offspring at PW2, PW12, PW28, and F2 male offspring at PW12 were fixed with 70% ethanol. Then, the bone mass was scanned and analyzed with the micro-CT system (VivaCT 40; Scanco, Basserdorf, Switzerland) as described previously^31^. To determine bone volume/total volume (BV/TV), trabecular number (Tb.N), trabecular thickness (Tb.Th) and trabecular separation (Tb.Sp), 0.5–5.5 mm below the lowest point of growth plate was selected as the region of interest and cross-sectional images were scanned at 21 μm resolution. These parameters of trabecular bone microarchitecture described above were computed using 3D model-independent algorithms (*n* = 8 per group).

### Cell culture and treatment

Bone marrow-derived mesenchymal stem cells (BMSCs) were obtained from 1-month-old rats. The cells were then collected and plated at a density of 4 × 10^5^ cells per well in six-well plates with growth medium (α-MEM with 10% fetal bovine serum, 100 mg/ml streptomycin and 100 U/ml penicillin). When the cells reached 80% confluence, growth medium was switched to osteogenic differentiation medium (α-MEM medium with 10% fetal bovine serum, 100 mg/ml streptomycin, 100 U/ml penicillin, 10 mM β-glycerophosphate, 50 μg/ml ascorbic acid and 10 nM dexamethasone). Then, the cells were treated with various concentrations of dexamethasone or co-treated with dexamethasone and captopril, RU486 or C646 for further analysis.

### siRNA knockdown of C/EBPα

To knockdown C/EBPα expression, RNA interference technology was used. The siRNA oligonucleotides against rat C/EBPα were purchased from Shanghai GenePharma Co. Ltd. (Shanghai, China). The sequences of the C/EBPα siRNA were CACGAGACGUCUAUAGACATT and UGUCUAUAGACGUCUCGUGTT. A pair of nonspecific oligonucleotides (nonsilencing control) was used as a negative control. The sequences of the control siRNA were UUCUCCGAACGUGUCACGUTT and ACGUGACACGUUCGGAGAATT. Prior to transfection, BMSCs were seeded in six-well plates at a density of 4 × 10^5^ cells per well. Twenty-four hours later, the cells were then transfected with 30 nM C/EBPα siRNA using Lipofectamine 2000 according to the manufacturer’s protocol. Eight hours later, the medium was exchanged for a fresh medium, and the cells were treated with dexamethasone. The cells were harvested for further analysis after 48 h.

### ELISA

To test the concentration of Ang II in bone tissue, equal weighting of bone tissue from each group was added 1 ml PBS for homogenate and centrifuged to collect supernatant. Then, the concentration of Ang II was measured by the Ang II ELISA kit following the manufacturer’s protocols. For testing the concentration of Ang II in cell culture supernatant, BMSCs were treated with dexamethasone to obtain supernatant from cell culture medium and used Ang II ELISA kit to detect the concentration of Ang II following the manufacturer’s protocols.

### Biochemical analysis of serum samples

The offspring were decapitated immediately to collect blood, and the serum samples were pooled and immediately frozen at −80 °C for subsequent analyses. The concentration of serum osteocalcin and TRAP activity were measured following the manufacturer’s protocols.

### Alizarin Red S staining

Mineralization was determined by Alizarin Red S staining as described previously^32^. The cultured cells were fixed in 95% ethanol for 30 min and then stained with 0.1% Alizarin Red S (pH 4.2) for 20 min at room temperature. They were then washed again with distilled water and observed by microscopy. Positive staining is represented as red color mineralization nodules.

### Total RNA extraction and RT-qPCR

Total RNA was isolated from bone tissue and BMSCs using TRIzol Reagent following the manufacturer’s protocol. The total RNA was reverse transcribed using a first strand cDNA synthesis kit. Then, RT-qPCR was performed using a SYBR Green qPCR Master Mix Kit and ABI StepOnePlus cycler (Applied Biosystems, Foster City, CA, USA) with 40 cycles. Relative expression of gene was calculated for each gene by the 2^-ΔΔCT^ method with glyceraldehyde 3-phosphatedehydrogenasefor normalization. The rat primer sequences for the genes used in this study were shown in Table S1.

### Western blotting

Cells were washed twice with ice-cold PBS and lysed in 200 μl RIPA Lysis Buffer and 1 mM phenylmethylsulfonyl fluoride for 5 min on ice to extract total protein. Cytoplasmic protein and nucleic protein were respectively extracted by the nuclear and cytoplasmic protein extraction kit following the manufacturer’s protocols. Equal amounts of protein lysates (30 μg per lane) were resolved by sodium dodecyl sulfate polyacrylamide gel electrophoresis on 10% polyacrylamide gels, transferred to polyvinylidene difluoride membranes and blotted with the primary antibodies for ACE (1:100 dilution), AT1R (1:500 dilution), AT2R (1:1000 dilution), GR (1:100 dilution), C/EBPα (1:500 dilution), or p300 (1:1000 dilution) at 4 °C overnight. Band intensity was quantified by using Quantity One (Bio-Rad, Shanghai, China).

### Chromatin immunoprecipitation (ChIP) assay

Cell suspensions from bone tissue and BMSCs were collected and fixed in 1% formaldehyde for chromatin cross-linking and added 125 mM glycine to stop the reaction. The samples were then centrifuged and resuspended in 0.5 ml lysis buffer containing protease inhibitors. Cell lysates were sonicated to shear DNA to lengths of ~ 200 base pairs and transferred to a new tube with ChIP dilution buffer. Chromatin was incubated overnight at 4 °C on nutator/rocker with specific antibody for H3K9ac (1:50 dilution) or H3K27ac (1:50 dilution) and BSA-treated Protein G beads to reduce nonspecific background binding. The immunoprecipitated DNA–protein complex with beads was collected by centrifugation and washed sequentially with low-salt, high-salt, LiCl immune complex, and Tris–ethylenediaminetetraacetic acid washing buffer solutions. Freshly prepared elution buffer (1% sodium dodecyl sulphate, 0.1 M NaHCO_3_) was used to elute the DNA–protein complex. The samples were then placed in 65 °C water baths overnight to reverse formaldehyde cross-linking and subsequently were purified using DNA purification kits. The isolated DNA was then assayed using RT-qPCR. The rat primer sequences of ACE promoter region in this study were as follows: forward, TCCACAAACACAACAGCTCG; reverse, AGAGAGGAGGAAGGTGGCTA.

### Data and statistical analysis

SPSS 20.0 (SPSS Science Inc., Chicago, Illinois, USA) and Prism 6.0 (GraphPad Software, La Jolla, CA, USA) were used to analyze experimental data. All data were expressed as mean ± standard error of the mean (S.E.M.). Student’s two tailed *t* test was performed on one factor of prenatal dexamethasone treatment (control or PDE). For the data from in vitro study, one-way analysis of variance followed by a post hoc Dunnett's *t* test or a post hoc Bonferroni *t* test were used to perform the multiple comparisons. A value of *P* < 0.05 was considered statistically significant.

## Results

### The low peak bone mass induced by PDE in F1 male offspring

**Fig. 2:**
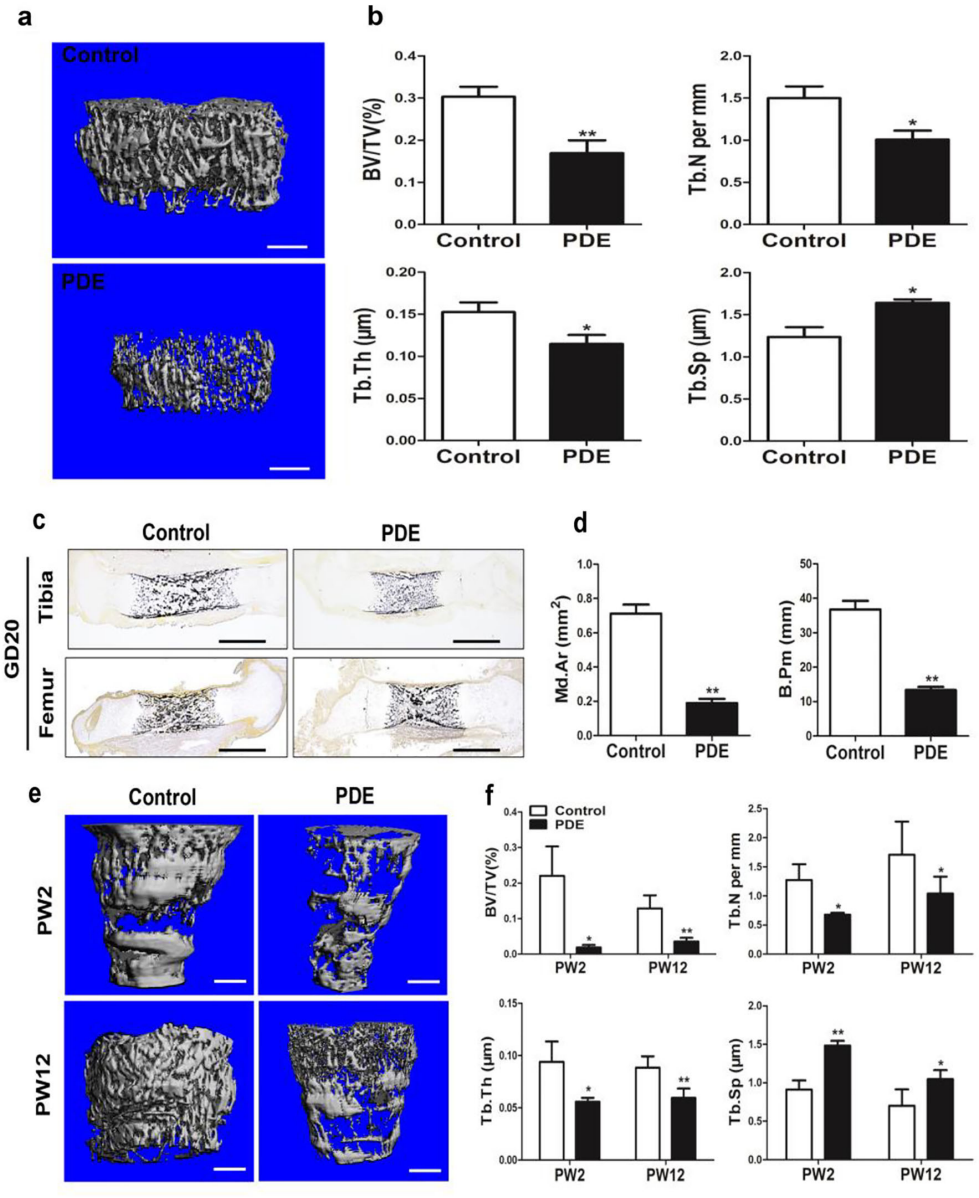
Effects of prenatal dexamethasone exposure (PDE) on bone mass in F1 male offspring. **a** Representative micro-CT images of femur from 28-week-old offspring with prenatal saline or dexamethasone treatment. **b** Quantitative micro-CT analysis of trabecular bone microarchitecture from 28-week-old offspring. **c** Representative von Kossa staining images of full-length tibia and femur from the control and PDE groups on gestation day (GD) 20. **d** Quantification of the mineralized area (Md.Ar) and bone perimeter (B.Pm) in primary ossification center. **e** Representative micro-CT images of femur from 2 or 12-week-old offspring with prenatal saline or dexamethasone treatment. **f** Quantitative micro-CT analysis of trabecular bone microarchitecture from 2 or 12-week-old offspring. Mean ± S.E.M., *n* = 8 per group, ^*^P < 0.05, ^**^P < 0.01 compared with the control. Scale bar = 500 μm. BV/TV, bone volume/trabecular volume; Tb.N, trabecular number; Tb.Th, trabecular thickness; Tb.Sp, trabecular separation

To explored the effect of PDE on peak bone mass, we measured the bone mass of F1 adult male offspring rats by micro-CT analysis. The results showed that PDE reduced the bone mass at PW28 (Fig. 2a), which was manifested as the decreased BV/TV, Tb.N and Tb.Th, and increased Tb.Sp (Fig. 2b). The above results demonstrated that PDE could lead to low peak bone mass in the offspring rats.

We then investigate whether the low peak bone mass induced by PDE was originated from fetal development. Thus, the effect of PDE on fetal bone mass was detected through von Kossa staining. The results showed that the Md.Ar and B.Pm in primary ossification center were significantly decreased by PDE, which indicated that PDE reduced the fetal bone mass in F1 male offspring (Fig. 2c, d). We also found that PDE significantly reduced the bone mass at PW12 (Fig. 2e), which was manifested as the decreased BV/TV, Tb.N and Tb.Th, and increased Tb.Sp (Fig. 2f). Moreover, the similar phenomenon was observed at PW2 (Fig. 2e, f). Collectively, these results indicated that PDE induced the low peak bone mass in the F1 male offspring, which was also originated from fetal development.

### The suppressed osteogenic differentiation and activated RAS induced by PDE in F1 male offspring

**Fig. 3:**
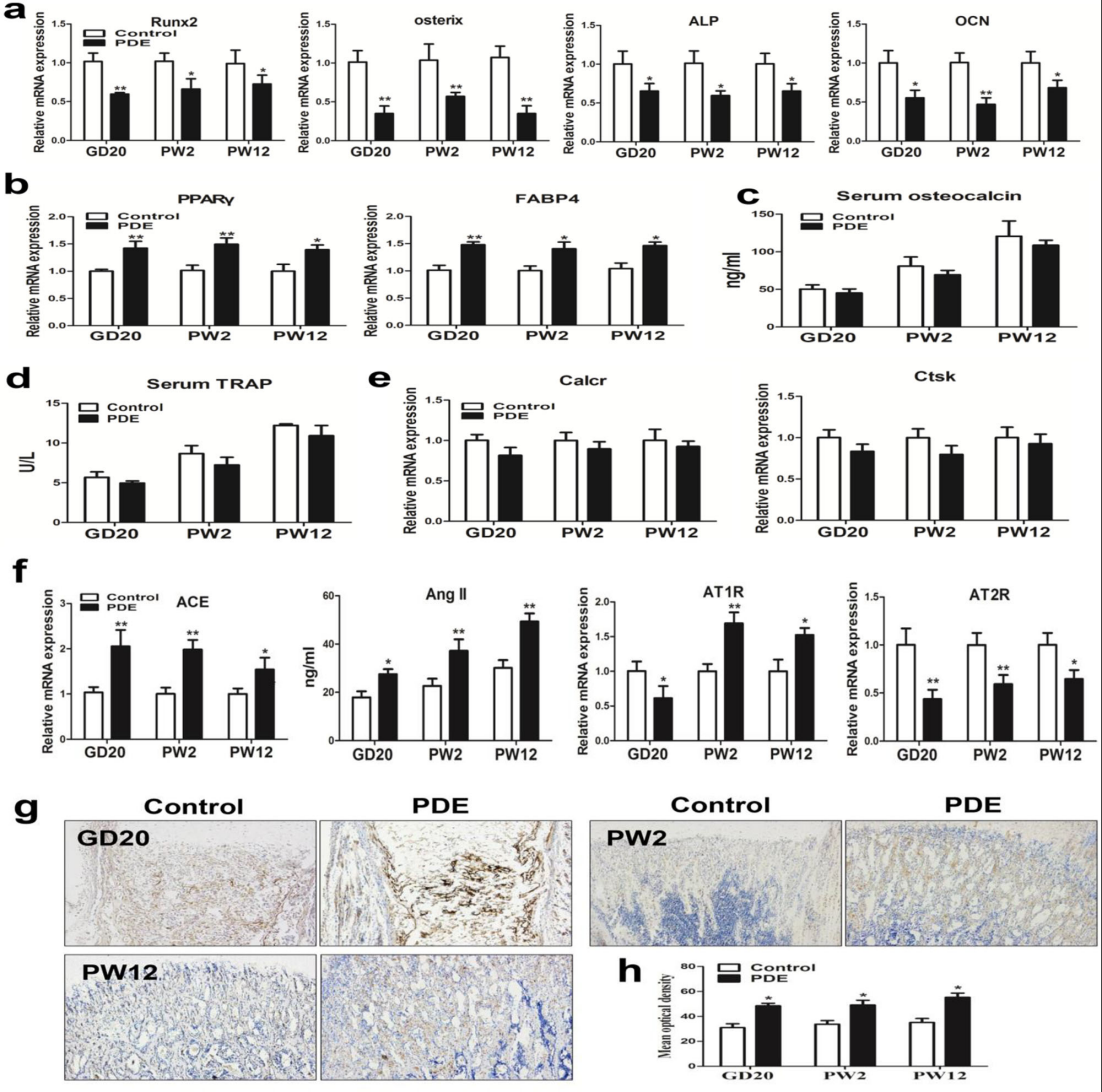
Effects of prenatal dexamethasone exposure (PDE) on osteogenic differentiation and local renin–angiotensin system (RAS) in F1 male offspring. **a** RT-qPCR analysis of gene expression of osteogenic differentiation markers, including Runx2, osterix, alkaline phosphatase (ALP), and osteocalcin (OCN) in bone tissue from gestational day (GD) 20 to postnatal week (PW) 12. **b** RT-qPCR analysis of gene expression of adipogenic differentiation markers, including peroxisome proliferator-activated receptor gamma (PPARγ) and fatty acid-binding protein 4 (FABP4) in bone tissue from GD20 to PW12. **c** ELISA analysis of serum osteocalcin from GD20 to PW12. **d** Analysis of serum TRAP activity from GD20 to PW12. **e** RT-qPCR analysis of gene expression of osteoclast differentiation markers in bone tissue from GD20 to PW12. **f** RT-qPCR analysis of gene expression of RAS, including angiotensin-converting enzyme (ACE), angiotensin receptors (ATRs), and ELISA analysis of angiotensin II (Ang II) production in bone tissue from GD20 to PW12. **g** Representative immunostaining images of ACE in bone tissue from GD20 to PW12. **h** Quantitative immunostaining analysis of the mean optical density of ACE from GD20 to PW12. Mean ± S.E.M., *n* = 8 per group, ^*^P < 0.05, ^**^P < 0.01 compared with the control

We then detected the expression of osteogenic differentiation markers in bone tissue, and found that the expression levels of Runx2, osterix, alkaline phosphatase (ALP) and OCN were all downregulated in the PDE group from GD20 to PW12 (Fig. 3a). Nevertheless, the adipocyte differentiation markers including peroxisome proliferator-activated receptor gamma (PPARγ) and fatty acid-binding protein 4 (FABP4) were all upregulated in the PDE group from GD20 to PW12 (Fig. 3b). These results suggested that PDE suppressed osteogenic differentiation but increased adipocyte differentiation potential continuously, which further contributed to the low peak bone mass in the F1 male offspring. Next, we detected serum turnover markers such as serum OCN to determine whether high level of bone turnover was involved in the decreased bone mass. The results showed that there was no significant difference in levels of serum OCN between the control and PDE offspring rats from GD20 to PW12 (Fig. 3c). We also measured serum TRAP and osteoclast differentiation markers to check whether the low peak bone mass is because of increased osteoclast number in bone tissue. The results indicated that the serum TRAP and osteoclast differentiation markers were all unaffected significantly by PDE from GD20 to PW12 (Fig. 3d, e). Taken together, the above results suggested that the low peak bone mass induced by PDE is because of suppressed osteogenic differentiation without the effects on increased bone turnover and osteoclast number.

Previous studies implied that the local RAS might involve in the process of cell differentiation^18^. Therefore, to investigate how PDE suppressed osteogenic differentiation in the F1 offspring, we detected the expression of RAS ingredients in bone tissue. The results indicated that PDE increased the expression of ACE and the production of Ang II from GD20 to PW12 (Fig. 3f). The expression of AT1R was decreased by PDE in GD20 but increased at PW2 and PW12 (Fig. 3f), whereas the expression of AT2R was decreased by PDE from GD20 to PW12 (Fig. 3f). Moreover, the immunohistochemical results suggested that the ACE protein expression in bone tissue was also increased by PDE from GD20 to PW12 (Fig. 3g, h). Overall, PDE could activate the local RAS in bone tissue sustainably by increasing ACE expression and Ang II production, while decreasing AT2R expression, which then suppressed osteogenic differentiation in the F1 male offspring.

### The intrauterine programming mechanism of the activated RAS induced by PDE in F1 male offspring

**Fig. 4:**
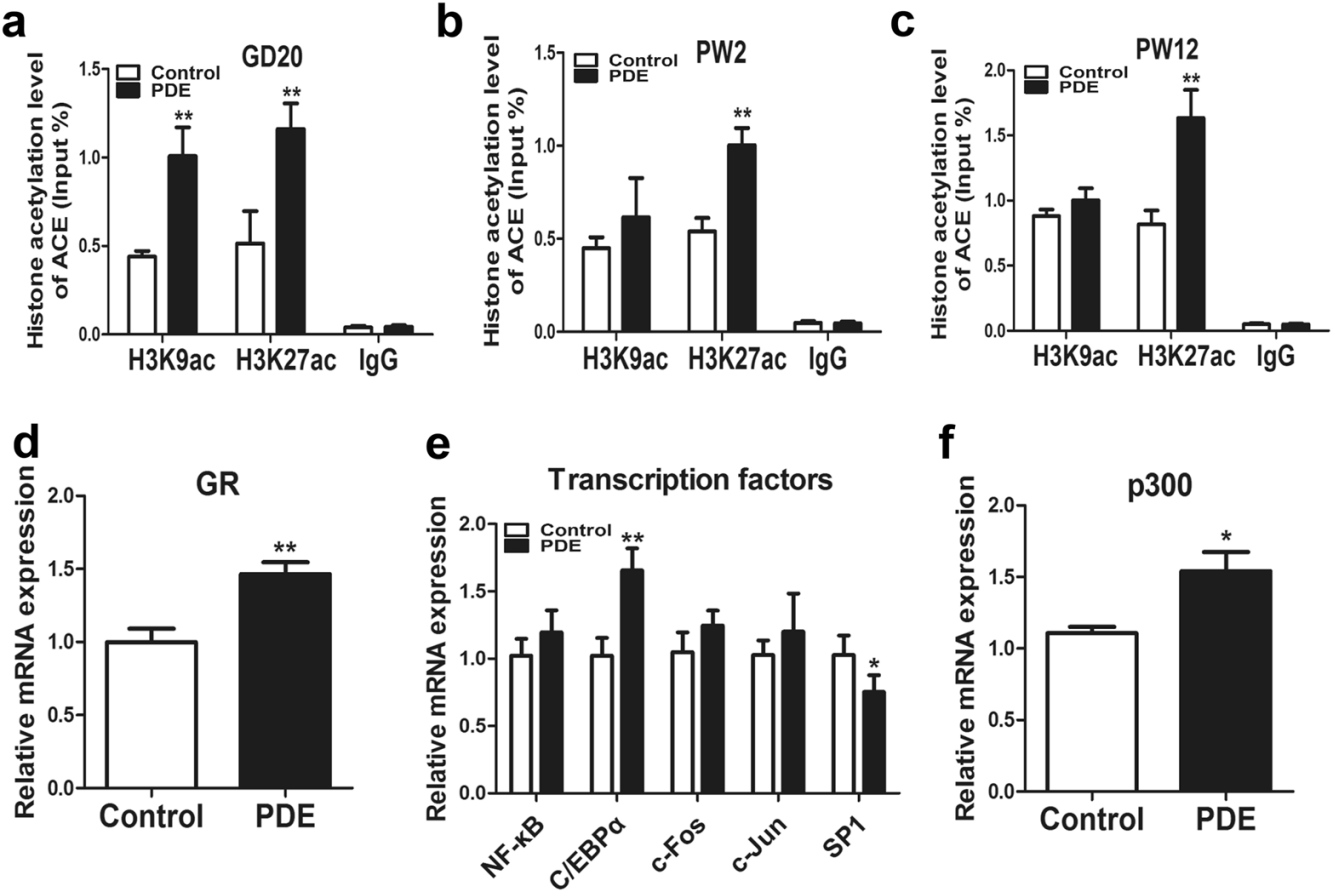
Effects of prenatal dexamethasone exposure (PDE) on the histone acetylation level of angiotensin-converting enzyme (ACE) and its intrauterine programming mechanism in F1 male offspring. **a** ChIP assay of the histone 3 lysine 9 acetylation (H3K9ac) and H3K27ac level in ACE promoter region on gestational day (GD) 20. **b** ChIP assay of the H3K9ac and H3K27ac level in ACE promoter region at postnatal week (PW) 2. **c** ChIP assay of the H3K9ac and H3K27ac level in ACE promoter region at PW12. **d** RT-qPCR analysis of glucocorticoid receptor (GR) expression in fetal bone tissue from the control and PDE groups. **e** RT-qPCR analysis of gene expression of transcription factors related to the function of GR, including nuclear transcription factor-κB (NF-κB), CCAAT/enhancer-binding protein α (C/EBPα), c-Fos, c-Jun, special protein 1 (SP1) in fetal bone tissue from the control and PDE groups. **f** RT-qPCR analysis of gene expression of p300 in fetal bone tissue from the control and PDE groups. Mean ± S.E.M., *n* = 8 per group, ^*^P < 0.05, ^**^P < 0.01 compared with the control

To investigate whether the epigenetic modification alteration induced by PDE participated in the sustained activation of local RAS from fetuses to adults, a ChIP assay was used to detect the histone acetylation level in ACE promoter region. And the results indicated that PDE markedly increased the H3K9ac and H3K27ac levels in the ACE promoter region on GD20 (Fig. 4a). Moreover, PDE only increased H3K27ac level of ACE, whereas there was no significant change in H3K9ac level at PW2 and PW12 (Fig. 4b, c). These findings suggested that PDE activated the local RAS continuously through increasing the H3K27ac level in ACE promoter region.

We further explored the potential mechanism about the increased H3K27ac level of ACE in response to PDE in fetuses and found that PDE significantly increased the expression of GR in fetal bone tissue (Fig. 4d). Moreover, we detected specific transcription factors that are closely related to the function of GR as described previously in fetal bone tissue^33^, including nuclear transcription factor-κB (NF-κB), c-Fos, c-Jun, C/EBPα, and special protein 1 (SP1). The results revealed that PDE failed to affect the expression of NF-κB, c-Fos, c-Jun, whereas the expression of SP1 was downregulated by PDE. Importantly, similar to the alteration of GR expression, PDE also upregulated the expression of C/EBPα (Fig. 4e). Furthermore, the expression of p300, as a crucial histone acetyltransferase, was increased by PDE (Fig. 4f). These above findings suggested that dexamethasone might recruit C/EBPα and p300 by activating GR, which cooperatively increased the H3K27ac level of ACE.

### The low peak bone mass induced by PDE in F2 male offspring and its intergenerational mechanisms

**Fig. 5:**
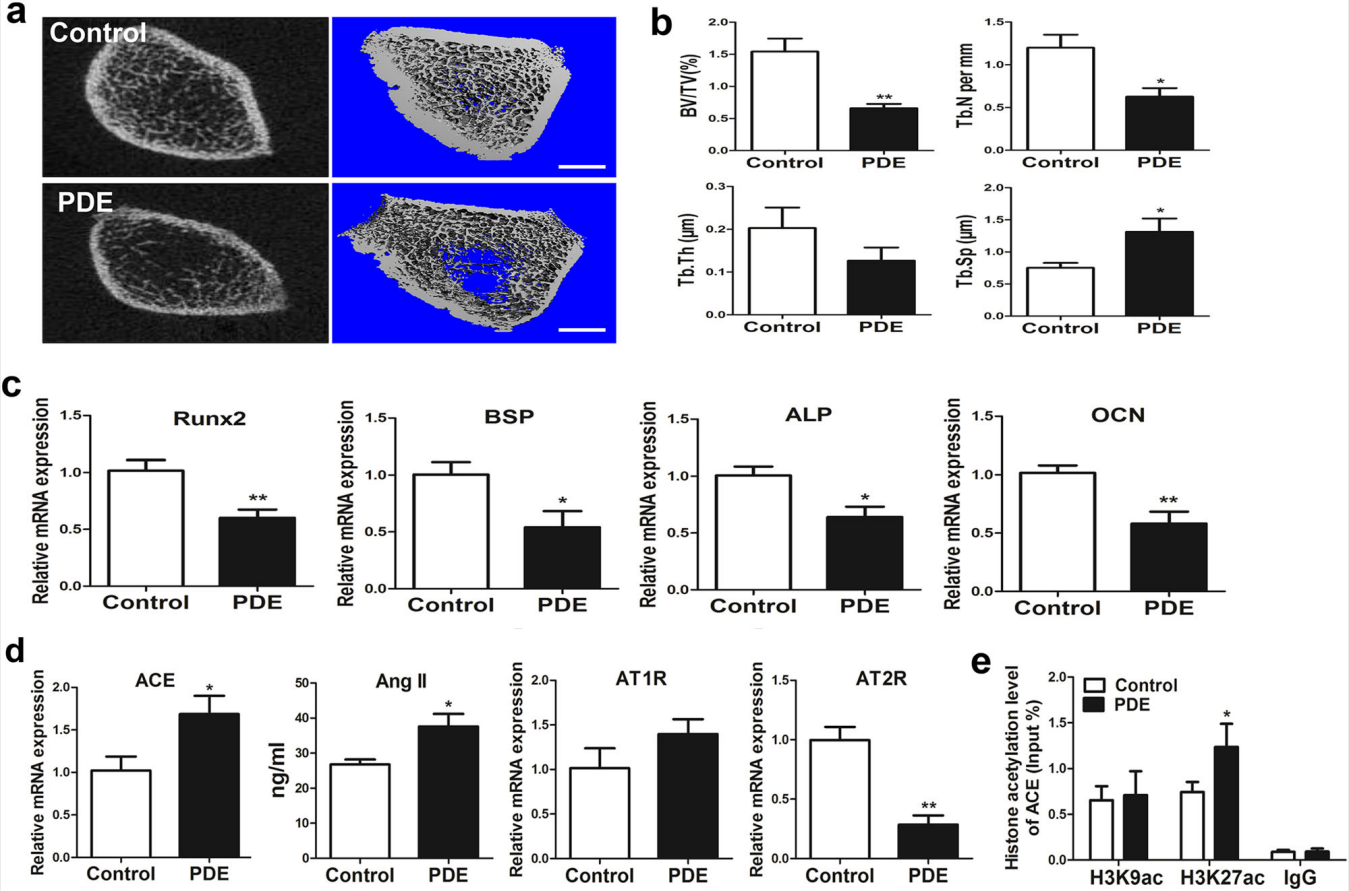
Effects of prenatal dexamethasone exposure (PDE) on the peak bone mass in F2 male offspring and its intergenerational mechanisms. **a** Representative micro-CT images of femur from F2 male offspring at postnatal week (PW) 12. **b** Quantitative micro-CT analysis of trabecular bone microarchitecture. **c** RT-qPCR analysis of gene expression of Runx2, bone sialoprotein (BSP), alkaline phosphatase (ALP), osteocalcin (OCN) from F2 male offspring at PW12. **d** RT-qPCR analysis of gene expression of renin–angiotensin system (RAS), including angiotensin-converting enzyme (ACE), angiotensin receptors (ATRs) and ELISA analysis of angiotensin II (Ang II) production in bone tissue from F2 male offspring at PW12. **e** ChIP assay of the histone 3 lysine 9 acetylation (H3K9ac) and H3K27ac level in ACE promoter region from F2 male offspring at PW12. Scale bar = 500 μm. Mean ± S.E.M., *n* = 8 per group, ^*^P *<* 0.05, ^**^P *<* 0.01 compared with the control

To determine whether the low peak bone mass induced by PDE has intergenerational effect, we analyzed the bone mass by micro-CT in F2 adult offspring. The results indicated that PDE reduced the bone mass in F2 male offspring (Fig. 5a), which was confirmed by the decreased BV/TV, Tb.N, and increased Tb.Sp (Fig. 5b). These results suggested that the low peak bone mass induced by PDE could transmit to F2 offspring. Moreover, the osteogenic differentiation markers were also downregulated by PDE in F2 offspring (Fig. 5c). We then found that the expression of ACE and the production of Ang II were significantly increased by PDE, but the expression of AT2R was decreased in response to PDE in F2 offspring. And there was no significant alteration of AT1R expression (Fig. 5d). These results indicated that the local RAS was also activated by PDE in F2 male offspring. Moreover, the H3K27ac level in ACE promoter region was significantly increased by PDE in F2 male offspring (Fig. 5e). Taken together, it suggested that the increased H3K27ac level of ACE mediated the intergenerational effect of low peak bone mass induced by PDE.

### The suppressed osteogenic differentiation and activated RAS induced by dexamethasone in BMSCs

**Fig. 6:**
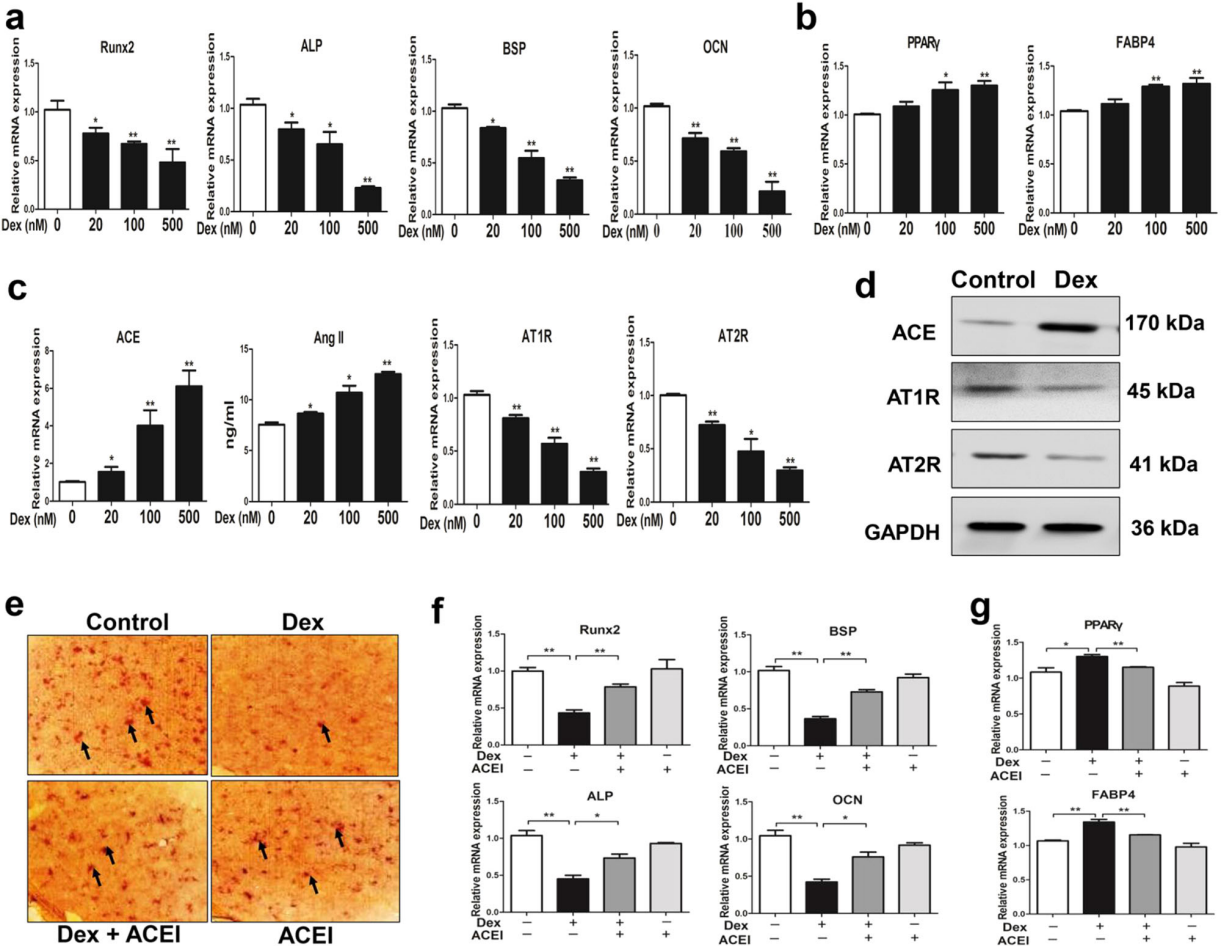
Effects of dexamethasone on osteogenic differentiation and renin–angiotensin system in bone marrow mesenchymal stem cells (BMSCs). **a** RT-qPCR analysis of gene expression of Runx2, alkaline phosphatase (ALP), bone sialoprotein (BSP), osteocalcin (OCN) in BMSCs cultured in the osteogenic medium and treated with different concentrations of dexamethasone for 14 days. **b** RT-qPCR analysis of gene expression of adipogenic differentiation markers, including peroxisome proliferator-activated receptor gamma (PPARγ) and fatty acid-binding protein 4 (FABP4) in BMSCs. **c** RT-qPCR analysis of gene expression of renin–angiotensin system (RAS), including angiotensin-converting enzyme (ACE), angiotensin receptors (ATRs), and ELISA analysis of angiotensin II (Ang II) production in BMSCs cultured in the osteogenic medium and treated with different concentrations of dexamethasone for 14 days. **d** Western blotting assay of ACE, AT1R, and AT2R protein level in BMSCs. **e** Alizarin Red S staining for mineralization nodules after co-treating BMSCs with dexamethasone and ACE inhibitor (ACEI) during the process of osteogenic differentiation. **f** RT-qPCR analysis of gene expression of Runx2, ALP, BSP, and OCN after co-treating BMSCs with dexamethasone and ACEI. **g** RT-qPCR analysis of gene expression of PPARγ and FABP4 after co-treating BMSCs with dexamethasone and ACEI. All experiments were performed at least three times. Mean ± S.E.M., ^*^P < 0.05, ^**^P < 0.01 compared with the untreated cells

To validate whether dexamethasone could inhibit osteogenic differentiation by activating RAS in vitro, BMSCs were treated with different concentrations of dexamethasone (20, 100, 500 nM) in the process of osteogenic differentiation culture. We found that the expression of osteogenic differentiation markers (Runx2, ALP, bone sialoprotein, and OCN) were downregulated with an average of 69% in high concentration of dexamethasone (500 nM) (Fig. 6a). However, the expression of adipocyte differentiation markers including PPARγ and FABP4 were upregulated with an average of 30% in high concentration of dexamethasone (500 nM) (Fig. 6b). Moreover, dexamethasone notably upregulated the mRNA expression of ACE and downregulated the mRNA expression of AT1R, AT2R. Meanwhile, the Ang II production was also increased by dexamethasone (Fig. 6c). Western blotting assay showed that the protein expression of ACE, AT1R, and AT2R were also consistent with their mRNA expression (Fig. 6d). We further co-treated BMSCs with dexamethasone and the ACE inhibitor captopril. Under this condition, by Alizarin Red S staining and osteogenic differentiation markers detection, we found that captopril alleviated the inhibitory effect of osteogenic differentiation and mineralization in response to dexamethasone (Fig. 6e, f). The ACE inhibitor captopril also partly reversed the increased adipocyte differentiation potential induced by dexamethasone (Fig. 6g). These above results indicated that dexamethasone mainly suppressed BMSCs osteogenic differentiation and mineralization by activating RAS, but increased adipogenic potential to some degree.

### The molecular mechanism of the activated RAS induced by dexamethasone

**Fig. 7:**
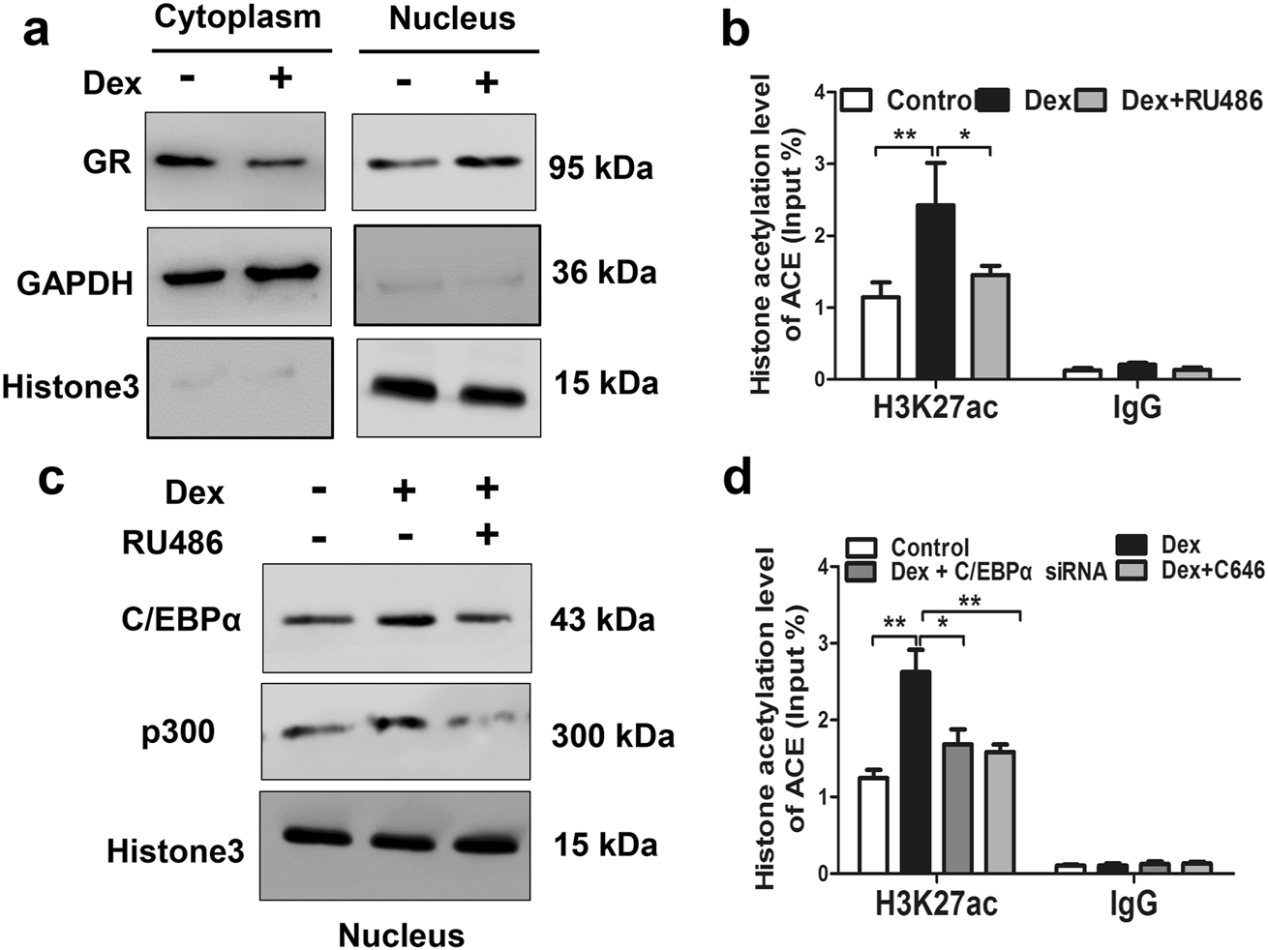
The molecular mechanism of the activated renin–angiotensin system (RAS) induced by dexamethasone in bone marrow mesenchymal stem cells (BMSCs). **a** Western blotting assay of glucocorticoid receptor (GR) protein level in cytoplasm and nucleus after treating BMSCs with dexamethasone. **b** ChIP assay of the histone 3 lysine 27 acetylation (H3K27ac) level in angiotensin-converting enzyme (ACE) promoter region in BMSCs treated with dexamethasone or co-treated with dexamethasone and GR inhibitor RU486. **c** Western blotting assay of CCAAT/enhancer-binding protein α (C/EBPα) and p300 protein level in nucleus after treating BMSCs with dexamethasone or co-treating BMSCs with dexamethasone and RU486. **d** ChIP assay of the H3K27ac level in ACE promoter region after co-treating BMSCs with dexamethasone and C/EBPα siRNA or p300 inhibitor C646. All experiments were performed at least three times. Mean ± S.E.M., ^*^P < 0.05, ^**^P < 0.01 compared with the untreated cells

Then, we verified the potential molecular mechanism about dexamethasone activating RAS in vitro. When treating BMSCs with dexamethasone in the process of osteogenic differentiation culture, we found that the GR protein level was decreased in the cytoplasm but increased in the nucleus (Fig. 7a). It demonstrated that dexamethasone could activate GR by promoting GR transfer into nucleus. What's more, the ChIP assay indicated that dexamethasone increased obviously H3K27ac level in the ACE promoter region. Whereas, the GR inhibitor RU486 alleviated the increased H3K27ac level induced by dexamethasone (Fig. 7b). Meanwhile, the protein level of C/EBPα and p300 in the BMSCs nucleus were also increased in response to dexamethasone, and RU486 partially reversed the above performance (Fig. 7c). Furthermore, the C/EBPα siRNA and p300 inhibitor C646 both alleviated the increased H3K27ac level of ACE induced by dexamethasone (Fig. 7d). Taken together, the above results indicated that dexamethasone recruited C/EBPα and p300 into nucleus via activating GR, which further cooperatively increased the H3K27ac level of ACE and activated RAS in the process of osteogenic differentiation.

## Discussion

### The low peak bone mass induced by PDE was originated from fetal development

Dexamethasone is exploited therapeutically in the perinatal period when there is a risk of preterm delivery to alter the rate of maturation of organs such as the lung. However, the application of dexamethasone during pregnancy could induce the toxic development of multiple organs^8,34,35^. Accumulating evidence from animal studies and clinical data also have raised concerns regarding the long-term consequences of prenatal dexamethasone treatment^36^. In this study, we treated pregnant rats with dexamethasone (0.2 mg/kg/d) during middle and late pregnancy to affirm the effect of PDE on peak bone mass. In combination with the dose conversion relationship between rats and humans (conversion coefficient is 6.16)^37^, prenatal treatment with dexamethasone in rats at 0.2 mg/kg/d in this study is comparable with that used in humans at 0.03 mg/kg/d. Owing to the standard dose of dexamethasone used in clinical is 0.05–0.2 mg/kg/d^38^, the exposure dose of dexamethasone in this study can be achieved in clinical and daily life.

As we known, peak bone mass is the maximum bone mass in humans or animals under physiological conditions. Moreover, the age of 7 ~ 9 months is the time when peak bone mass occurs in rats, and the age of 6 ~ 12 weeks in rats is the critical period to achieve peak bone mass in the whole life. In recent research, we showed that PDE reduced the offspring’s bone mass at PW28 (the age of 7 months old), the results demonstrated that PDE could lead to low peak bone mass in the offspring rats. Moreover, the result indicated that the fetal bone mass in primary ossification center was decreased by PDE. Also, PDE significantly reduced the bone mass at PW12 with the decreased BV/TV, Tb.N, Tb.Th, and increased Tb.Sp. Accordingly, our data demonstrated that PDE could lead to the low peak bone mass in the F1 adult male offspring, which was originated from fetal development. As the peak bone mass contributes to 60% of the osteoporosis risks^12^, the low peak bone mass induced by PDE even may lead to osteoporosis in old age. Thus, from a clinical point of view, it is necessary to applicate dexamethasone properly during pregnancy.

### The sustained activation of RAS caused by dexamethasone mediates osteogenic differentiation inhibition and low peak bone mass

BMSCs have multiple differentiation potential and can be differentiated into osteoblasts, chondrocytes, and adipocytes^15^. In the process of osteogenic differentiation, BMSCs first differentiated into osteoblast progenitor cells and then migrated to the bone surface to further differentiate into mature osteoblasts. Osteogenic differentiation potential of BMSCs has a vital role in the process of endochondral ossification and is one of the important factors to maintain bone mass homeostasis^39^. Thus, we explored the effects of PDE on osteogenic differentiation in F1 offspring. Our results showed that the osteogenic differentiation markers, such as Runx2, osterix, ALP, and OCN were both decreased by PDE, but the adipocyte differentiation markers, including PPARγ and FABP4 were upregulated by PDE from GD20 to PW12 in F1 male offspring. When treating BMSCs with different concentrations of dexamethasone, we also found that high concentration of dexamethasone could inhibit the expression of osteogenic differentiation markers with an average of 69%. However, the expression of adipocyte differentiation markers was upregulated on average 30% by dexamethasone. These results demonstrated that the reduction of peak bone mass induced by PDE was mainly due to the sustained suppression of osteogenic differentiation, but increased adipogenic potential to some degree.

Previous studies have suggested that cell differentiation could be modulated by the local RAS^20,40^. Then, we detected the expression of RAS ingredient in bone tissue and found that the local RAS in bone tissue was activated by PDE from GD20 to PW12, mainly manifested as the increased ACE expression and Ang II production but decreased AT2R expression. These results suggest that the increased expression of ACE mediated the sustained activation of RAS in local bone tissue, which further leads to osteogenic differentiation suppression. Meanwhile, we have found that the ACE inhibitor captopril could rescue the suppressed effect of osteogenic differentiation caused by dexamethasone in vitro, which further demonstrates that dexamethasone inhibited BMSCs osteogenic differentiation by activating RAS. Altogether, this study indicated that the activated local RAS mediates the suppression of osteogenic differentiation and low peak bone mass induced by dexamethasone, and ACE could become an early therapeutic target.

### The abnormal epigenetic modification of ACE resulting from dexamethasone mediates the intrauterine programming alteration of RAS

Adverse environment during pregnancy, such as prenatal xenobiotic exposure, can influence the phenotype of offspring constantly through epigenetic modification regulation^41,42^. More and more studies have shown that the epigenetic modification has an important role in the process of intrauterine programming^25,27^. Synthetic glucocorticoids, including dexamethasone, also could lead to alterations in epigenetic modifications, expression, and function of many target genes, having an important role in intrauterine programming mechanism of diseases^28^. Thus, when investigating the mechanism of sustained activation of local RAS programed by PDE, we detected the histone acetylation level of ACE in bone tissue from GD20 to PW12. The results indicated that the increased level of H3K27ac in the ACE promoter region mediated the local RAS activation induced by dexamethasone.

We further explored the potential mechanism of the increased H3K27ac level in ACE promoter region caused by dexamethasone. Previous researches suggest that glucocorticoids mainly play a role through GR in regulating the expression of target genes. GR is one of the member of nuclear receptors, which once activated usually recruit transcription factors (such as NF-κB, c-Fos, c-Jun, C/EBPα, SP1) and epigenetic modification enzyme into the nucleus, altering the epigenetic modifications of target genes^33^. So, we detected the GR expression in fetal bones and found that PDE could promote GR expression. Meanwhile, we found that the expression of C/EBPα increased significantly. Moreover, the expression of p300 also increased in fetal bone tissue. Then, we confirmed the above mechanism in vitro, and found that dexamethasone could increase the C/EBPα and p300 protein level in BMSCs nucleus by activating GR. Meanwhile, the application of GR inhibitor, C/EBPα siRNA, and p300 inhibitor could partially reverse the increased H3K27ac level of ACE induced by dexamethasone. Taken together, these data indicated that dexamethasone recruited C/EBPα and p300 into nucleus by activating GR, and then cooperatively increased the H3K27ac level of ACE, which further mediated the sustained activation of local RAS.

### The intrauterine programming alteration of RAS mediates the intergenerational effect of low peak bone mass induced by dexamethasone

Increasing evidences indicated that environmental events during pregnancy and early postnatal stage could affect histone acetylation of promoter region of specific genes, which then not only maintained throughout the life but stably transmitted to the next generation^43,44^. For example, Iqbal et al.^45^ reported a similar alteration of the hypothalamic–pituitary–adrenal axis and animal behavior in both juvenile and adult guinea pigs after maternal betamethasone exposure, which also is characterized by an inheritable phenomenon. The environmental influence on pregnant mothers is not limited to the mothers itself but is also directly related to the fetus (F1) and its germline, which finally leads to associated phenotype alteration in the F2 offspring^43,46^. The present study demonstrated, for the first time, that PDE lead to the low peak bone mass in F2 male offspring. We further found that the H3K27ac level in the ACE promoter region was increased by PDE in the F2 offspring. Thus, our data suggested that the increased H3K27ac level of ACE contributed to the intergenerational effect of low peak bone mass induced by dexamethasone. Recent studies indicate that when a programming intervention is applied to a mother (F0) during pregnancy, it will directly influence the offspring developing in utero (F1)^47^. However, the germ cells (future gametes) that will form the F2 offspring develop during this pregnancy, and hence will be directly exposed to the adverse environment^48^. Thus, we consider that the increased H3K27ac level of ACE in the F2 offspring induced by dexamethasone might be from the sperm cells in F1 offspring and then lead to the low peak bone mass in F2 offspring.

### Summary

**Fig. 8:**
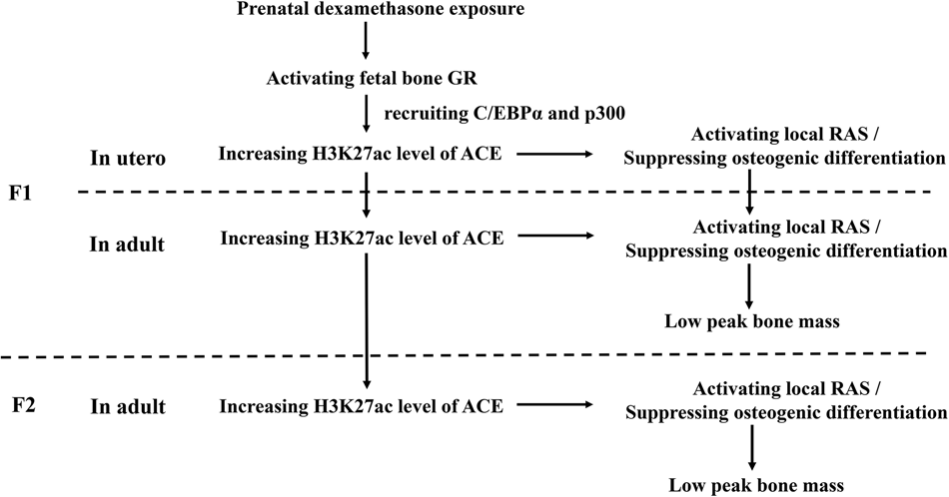
Increased H3K27ac level of ACE mediates the intergenerational effect of low peak bone mass induced by prenatal dexamethasone exposure in male offspring rats. GR, glucocorticoid receptor; C/EBPα, CCAAT/enhancer-binding protein α; ACE, angiotensin-converting enzyme ACE; H3K27ac, histone 3 lysine 27 acetylation; RAS, renin–angiotensin system

In this study, we confirmed that the low peak bone mass induced by PDE has intergenerational effect. The intrauterine programming mechanism is that dexamethasone recruited C/EBPα, p300 into nucleus through activating GR, which cooperatively increased H3K27ac level in the ACE promoter region. And this increased H3K27ac level of ACE continued from F1 offspring to F2 offspring, which further contributed to the sustained activation of RAS and suppressed osteogenic differentiation in the abovementioned offspring (as shown in Fig. 8). This study provides new experimental evidences for the intrauterine origin, intergenerational inheritance and therapeutic targets of bone developmental toxicity.

## Electronic supplementary material

Table S1. Primers used in quantitative real-time PCR

Supplementary table 1 legend
