# Supplementary material for: Increased H3K27ac level of ACE mediates the intergenerational effect of low peak bone mass induced by prenatal dexamethasone exposure in male offspring rats
Source: Cell Death Dis. 2018 May 29;9(6):638. doi: 10.1038/s41419-018-0701-z (PMC5974192; doi:10.1038/s41419-018-0701-z)
Supplement: Supplementary file 1 — Table S1. Primers used in quantitative real-time PCR [file 41419_2018_701_MOESM1_ESM.docx]

**Table S1. Primers used in quantitative real-time PCR**

| Genes | Forward primers | Reverse primers | Annealing |
| --- | --- | --- | --- |
| Runx2 | TACTTCGTCAGCGTCCTATC | CAGCGTCAACACCATCATT | 60 °C, 30 s |
| osterix | GGAAAGGAGGCACAAAGAAGC | CCCCTTAGGCACTAGGAGC | 58 °C, 20 s |
| ALP | CGTCTCCATGGTGGATTATG | GGATGTAGTTCTGCTCATGG | 58 °C, 20 s |
| BSP | GAGTACAACACTGCGTATGA | GTAATAATCCTGACCCTCGTAG | 58 °C, 20 s |
| OCN | CAGACCTAGCAGACACCATG | GCTTGGACATGAAGGCTTTG | 60 °C, 30 s |
| PPARγ | TGTGGACCTCTCTGTGATGG | CATTGGGTCAGCTCTTGTGA | 60 °C, 30 s |
| FABP4 | ACATGAAAGAAGTGGGAGTTGGC | AAGTACTCTCTGACCGGATGACG | 60℃, 30 s |
| Calcr | CGTGCCGTCTACTACAACGA | AGAAGTTGACCACCAGAGCC | 60℃, 30 s |
| Ctsk | GACCCGTCTCTGTGTCCATC | ACGGTCGCAGTTTTCGTCAT | 60℃, 30 s |
| ACE | CGTCAACTTCCTGGGTATG | GGAGGCTGTGATGGTTATG | 58 °C, 20 s |
| AT1R | CTCTGTTCTACGGCTTTCTG | GAGCTCATGTTATCCGAAGG | 58 °C, 20 s |
| AT2R | GAGAAATATGCTCAGTGGTCTG | GACTTGGTCACGGGTAATTC | 60 °C, 30 s |
| GR | CCAGGCTTCAGAAACTTACA | CATGCAGGGTAGAGACATTC | 60 °C, 30 s |
| NF-κB | CGACGTATTGCTGTGCCTTC | TTGAGATCTGCCCAGGTGGTA | 58 °C, 20 s |
| C/EBPα | CGCAAGAGCCGAGATAAAG | TTGACCAAGGAGCTCTCA | 60℃, 30 s |
| c-Fos | GGGAGCTGACAGATACGCTC | TTGGCAATCTCGGTCTGCAA | 60 °C, 30 s |
| c-Jun | TGCAAAGATGGAAACGACCTT | GCCGTAGGCGCCACTCT | 60 °C, 30 s |
| SP1 | AGGATGCGGCAAAGTATATG | GCTCATCCGAACGAGTAAAG | 60 °C, 30 s |
| p300 | CAAATGCAGGCATGGGCAAT | TCCTGGTTGTCCTCCCATCT | 60 °C, 30 s |
| GAPDH | GCAAGTTCAACGGCACAG | GCCAGTAGACTCCACGACA | 60 °C, 30 s |
