# Supplementary material for: Increased H3K27ac level of ACE mediates the intergenerational effect of low peak bone mass induced by prenatal dexamethasone exposure in male offspring rats
Source: Cell Death Dis. 2018 May 29;9(6):638. doi: 10.1038/s41419-018-0701-z (PMC5974192; doi:10.1038/s41419-018-0701-z)
Supplement: Supplementary file 2 — Supplementary table 1 legend [file 41419_2018_701_MOESM2_ESM.docx]

**Supplemental table S1** **Primers used in quantitative real-time PCR.** ALP, alkaline phosphatase; BSP, bone sialoprotein; OCN, osteocalcin; PPARγ, peroxisome proliferator-activated receptor γ; FABP4, fatty acid-binding protein 4; Calcr, calcitonin receptor; Ctsk, cathepsin K; ACE, angiotensin converting enzyme; AT1R, angiotensin receptor 1; AT2R, angiotensin receptor 2; GR, glucocorticoid receptor; NF-κB, nuclear transcription factor-κB; C/EBPα, CCAAT/enhancer -binding protein α; SP1, special protein 1; GAPDH, glyceraldehyde-3-phosphate dehydrogenase.
